# Simple and Visible Detection of Novel Astroviruses Causing Fatal Gout in Goslings Using One-Step Reverse Transcription Polymerase Spiral Reaction Method

**DOI:** 10.3389/fvets.2020.579432

**Published:** 2020-12-10

**Authors:** Jun Ji, Qinxi Chen, Zhengli Yu, Xin Xu, Xinhao Mu, Xiang Tian, Xiaoge Fu, Lunguang Yao, Yingzuo Bi, Qingmei Xie

**Affiliations:** ^1^Henan Provincial Engineering Laboratory of Insects Bioreactor, Henan Provincial Engineering and Technology Center of Health Products for Livestock and Poultry, Henan Provincial Engineering and Technology Center of Animal Disease Diagnosis and Integrated Control, Nanyang Normal University, Nanyang, China; ^2^College of Animal Science, South China Agricultural University, Guangzhou, China

**Keywords:** N-GoAstV, rapidity, simplicity, visible detection, RT-PSR

## Abstract

In this study, a one-step isothermal method combining polymerase spiral reaction (PSR) with reverse transcription (RT-PSR) was established for rapid and specific detection of novel astroviruses causing fatal gout in goslings (N-GoAstV). The one-step RT-PSR was accomplished at the optimal temperature of 62°C and time of 40 min and used primers simply designed as conventional PCR primers, and the results of detection were visible to the naked eye. The detection limit of PSR was above 34.7 copies/μL at a 95% probability level according to probit regression analysis. The assay specifically detected N-GoAstV, and no other reference viruses were detected. These results suggest that the newly established RT-PSR assay could, in one step, accomplish reverse-transcription, amplification, and result determination providing a visible, convenient, rapid, and cost-effective test that can be carried out onsite, in order to ensure timely quarantine of N-GoAstV-infected birds, leading to effective disease control.

## Introduction

Astroviruses (AstV) are small non-enveloped RNA viruses that can infect many hosts, including humans (1), livestock, poultry, domestic pets (2), even bats (3), and aquatic birds (4). Since February 2016, some goose farms in the Shandong Province of China experienced an outbreak of disease characterized by hyperuricemia and urate depositions to the viscera and joint capsules and even under the skin of goslings at 1 to 3 weeks of age. This was diagnosed as gout (5, 6). The disease impacted the birds' motor functions and feed intake, leading to emaciation and death. The cause of the gout outbreak in goslings had been confirmed to be novel goose astrovirus (N-GoAstV) (6, 7). An increasing number of cases of astrovirus-infected goslings developing gout has been reported since December 2017 (6), and this condition occurs persistently in some provinces, causing huge economic losses to the poultry industry (7–9). Therefore, effective control of these N-GoAstV infections and the development of diagnostic methods for simple, rapid, user-friendly, and efficient detection of N-GoAstV are imperative.

Traditional immunological methods are not ideal for diagnosing novel pathogeny due to the complex preparation required and the need to screen out highly specialized antibodies in the serum. Various molecular-based methods, including reverse transcription-polymerase chain reaction (RT-PCR) (5) and reverse transcription quantitative PCR (RT-qPCR) (10, 11) have been successfully implemented to diagnose N-GoAstV in clinical samples. Despite the fact that these methods offer rapidity, specificity, and sensitivity, they cannot be widely used in the field because of the sophisticated procedures involved and the requirement for expensive instrumentation (12). To solve these problems of detection, assays based on PCR technology and loop-mediated isothermal amplification (LAMP) have been implemented for N-GoAstV detection (13). Additionally, a novel nucleic acid amplification method called polymerase spiral reaction (PSR) has also been developed to amplify the target sequence within a short time (usually 1 h or less) under isothermal conditions (60°C−65°C) (14). Recently, the PSR assay or reverse transcription PSR (for pathogens with RNA genomes) has been extensively used for the identification of bacteria, viruses, and even for molecular pathology analyses (15–17). Hence, we developed a one-step RT-PSR method for detecting the N-GoAstV in this study.

## Methods and Materials

### Viruses and Clinical Samples

From 2018 to 2019, spleen, liver, and kidney tissues of dead goslings suspected to have been infected by N-GoAstV were collected from goose farms in central China. These tissue samples were washed several times with phosphate-buffered saline (PBS) to eliminate residual blood and then placed in a centrifuge tube and stored at −80 °C until required.

The homogenized tissues collected from the goslings described above were frozen and thawed three times, treated with 200 μg/ml gentamicin and 200 μg/ml penicillin, and incubated for 3. The samples were then centrifuged at 7,000 × g and inoculated onto the monolayers of Leghorn Male chicken Hepatocellular-carcinoma cell lines (LMH), and the virus was passed in cell culture subsequently determined by more propagation within five generations. After five passages, N-GoAstV was confirmed by RT-PCR according to Zhang, et al. (18). Meanwhile, N-GoAstV, goose parvovirus (GPV), goose reovirus (GREOV), goose hemorrhagic polyomavirus (GHPV), and Tembusu virus (TMUV) strains were stored in the Henan Provincial Engineering and Technology Center of Health Products for Livestock and Poultry, Nanyang Normal University.

### Total Viral DNA/RNA Extraction

Total viral DNAs and RNAs of the clinical samples and culture lysates of virus isolation were extracted using extraction kits (EasyPure Viral DNA/RNA Kit; TransGen Biotechnology, Inc., Beijing, China) according to the instruction book. The purity and concentrations of the DNA/RNA samples were determined by biological spectrophotometry, and the viral DNA/RNAs were stored at −80°C until required.

### Primer Design for RT-PSR

The spiral primers (SF and SR) used for the RT-PSR assays were designed using Primer Premier 5 (PREMIER Biosoft International Co., United States) based on the frequently conserved ORF1b gene in the genome of N-GoAstV (Table 1).

**Table 1:** Primer sets for one-step RT-PSR (The lower-case 5′ sequence of the forward primer (SF) abstracted from a botanic gene is the reverse of lowercase 5′ sequence of the reverse primer (SR) (14).

**Primer name**	**Sequence 5^**′**^-3^**′**^**	**Position within GoAstV-*ORF*1b**
SF	acgattcgtacatagaagtatagTATGATCTTGTGTGCT*R*^a^ATCCAGT	606–629
SR	gatatgaagatacatgcttagcaACCACCAATGAGCCTAGATACTCG	745–768

a *Italic text indicates degenerate oligonucleotides, R = A + G*.

### Establishing the Reaction Temperature and Time for One-Step-RT-PSR

The one-step RT-PSR was carried out at a final reaction volume of 25 μL, including 2 mM dNTPs, 8 mM MgSO_4_, 10 mM (NH_4_)_2_SO_4_, 100 mM KCl, 0.1% v/v Tween-20, 8 U *Bst* DNA polymerase 3.0 (New England Biolabs, Hitchin, UK), primer SF and SR (both 1.6 μM), 1 μL of total RNA template (~ 10 ng /μL). The reaction mixture was further added 1 μL pH-sensitive indicator (comprising 0.08 mM cresol red and 0.025 mM phenol red) for visible interpretation and mineral oil to prevent the volatilization of the RT-PSR products. The one-step RT-PSR was conducted at a temperature gradient of 60−65°C for 60 min to determine the optimum reaction temperature. The optimum time for the one-step RT-PSR, evaluated based on the optimum temperature over a time gradient of 10–60 min for Bst 3.0 DNA polymerase, has improved isothermal amplification performance and strong reverse transcription; the addition of exogenous reverse transcriptase and reverse transcription is not required. The products of one-step RT-PSR were electrophoresed on 2% agarose gel in 1 × TAE buffer and directly visualized under natural light by color change (yellow indicated a positive result, but purple-red indicated a negative result).

### Sensitivity and Specificity of the One-Step RT-PSR Assay

In order to compare assays, a RT-LAMP reaction assay was also performed using a water bath at 62°C for 30 min, as previously described (13). To analyze the sensitivity of the one-step RT-PSR, the standard plasmid (pT-ORF1b, harbored ORF1b gene of AstV/HN01/Goose/0103/18 strain) preserved in our lab and the copy number were calculated according to the following formula: (copies/μL = 6.02 × 10^23^ × DNA concentration, g/μL)/molecular weight, g/mol, as described by Ji et al. (13). Ten-fold serial dilutions of pT-ORF1b in nuclease-free water (diluted from 3.47 × 10^0^) were subjected to the newly developed one-step RT-PSR amplification as well as to the RT-LAMP amplification in 20 replicates. The products of the one-step RT-PSR and one-step RT-LAMP were assayed by 2.0% agarose gel electrophoresis.

The specificity of the one-step RT-PSR primers [synthesized at Hongxun Biotechnologies Ltd. (Suzhou, China)] were examined using preserved N-GoAstV, GPV, GREOV, GHPV, and TMUV, and nuclease-free water was used as the negative control. In addition, the ORF1b genes of other goose-origin astroviruses [FLX strain, Accession no. (KY271027) and AHDY strain, Accession no. (MH410610)] were also used in the specificity testing. All the reference pathogen or template were detected by one-step RT-PSR in 20 replicates.

### Validation With Clinical Samples

A total of 235 clinical samples from geese with gout described above were used to further evaluate the one-step RT-PSR assay. Both the one-step RT-PSR and one-step RT-LAMP assays were performed to compare the positive rates using virus isolation as a gold standard. All products of one-step RT-PSR were visualized by adding phenol red and cresol red staining.

The samples tested positive for one-step RT-PSR assay were further screened for the presence of N-GoAstV by PCR using a 20-μL reaction mixture containing a DNA template (amplification products of one-step RT-PSR assay), including 10-pmol primers (SF and SR), Ex-Taq DNA polymerase (TaKaRa Biotechnology Co., Ltd., Dalian, China) and nuclease-free water. The obtained amplicons were subsequently sequenced (Syn-Biotechnology, Suzhou, China).

#### Statistical Analysis

A probit analysis to determine the detection limit of one-step RT-PSR was performed at a 95% probability level, and the κ (kappa) and *p*-values of virus isolation and one-step RT-PSR were calculated. The sensitivity and specificity testing for detection evaluation were also calculated. All statistical analyses were performed in SPSS 25.0 (IBM Corporation, New York, United States).

## Results

### Optimal Reaction Temperature and Time for One-Step RT-PSR Assay of N-GoAstV

As shown in Supplementary Figure 1, in the agarose gel electrophoresis analysis, little difference was observed in the gradient bands produced over the temperature range from 60°C to 65°C. Because of the brightness of the one-step RT-PSR products and reaction median, 62°C was chosen as the optimal reaction temperature. Subsequently, no bands appeared in the first 10 min at 62°C, but the bands slowly grew brighter as time went on, and brightness peaked after 40 min. Thus, the optimal one-step RT-PSR assay reaction conditions for N-GoAstV detection were 62°C and 40 min.

### Determination of the One-Step RT-PSR Sensitivity

**Figure 1:**
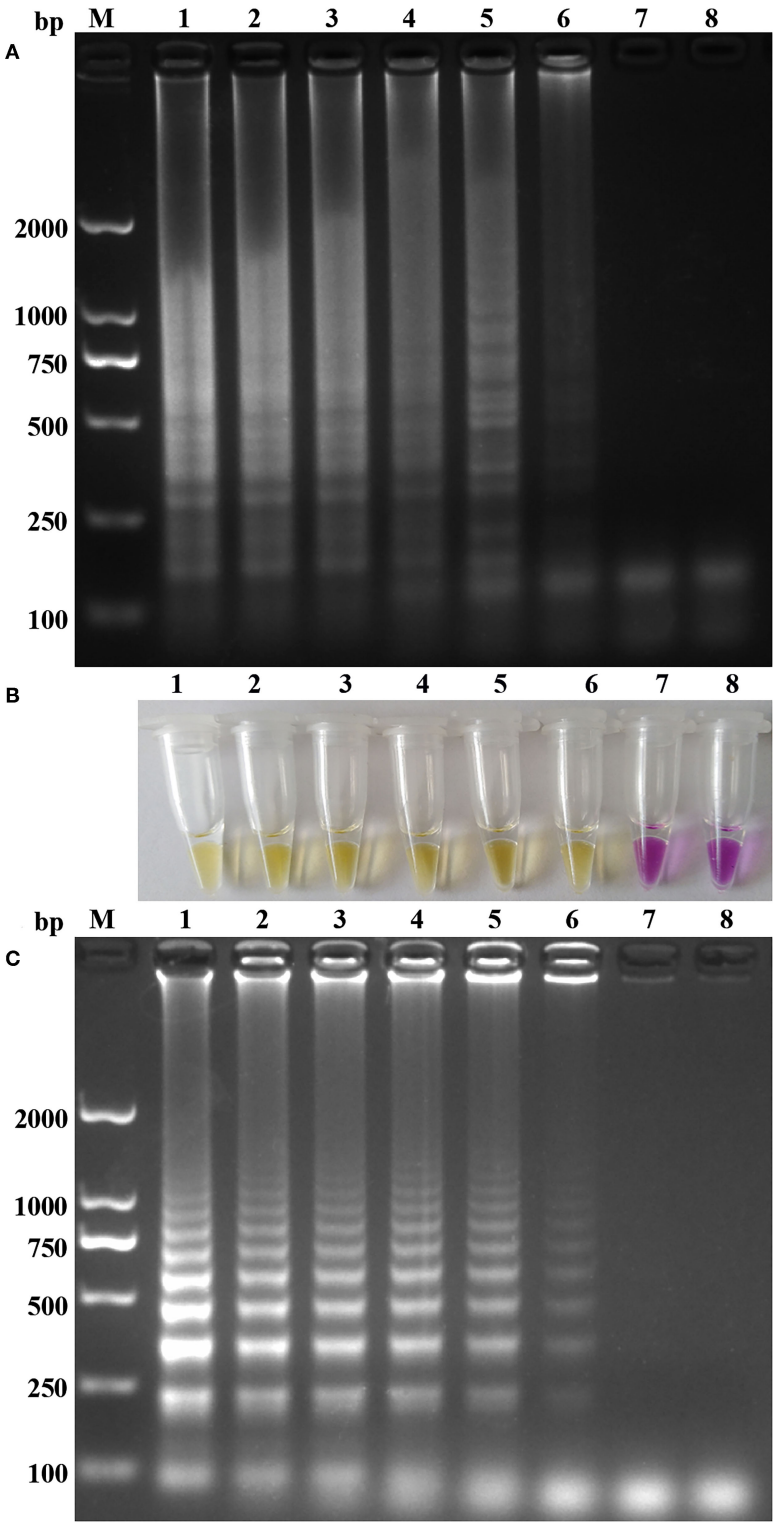
Sensitivity of the one-step RT-PSR amplification for N-GoAstV: **(A,C)** Agarose gel electrophoresis of RT-PSR and RT-LAMP amplification products. **(B)** Visual detection of positive and negative RT-PSR amplification products. Lane M, molecular size marker DL2000; Lanes1-7, DNA template with 3.47 × 10^6^ to 10^0^ copies; Lane 8, negative control.

As shown in Figure 1, the sensitivities of the one-step RT-PSR were compared with one-step RT-LAMP assays by the color change and agarose gel electrophoresis of their reaction products. All dilutions of the pT-ORF1b from 3.47 × 10^7^ to 10^1^ copies per reaction were positive in the assays. Only one of the twenty replicates with 3.47 copies tested positive in the RT-PSR assay (displayed in Supplementary Table 1). The positive tube indicated that the detection limit of the one-step RT-PSR was 34.7 copies/μL (probit analysis, *p* < 0.05), which was similar with that of the RT-LAMP previously reported (13).

### Determination of the One-Step RT-PSR Specificity

**Figure 2:**
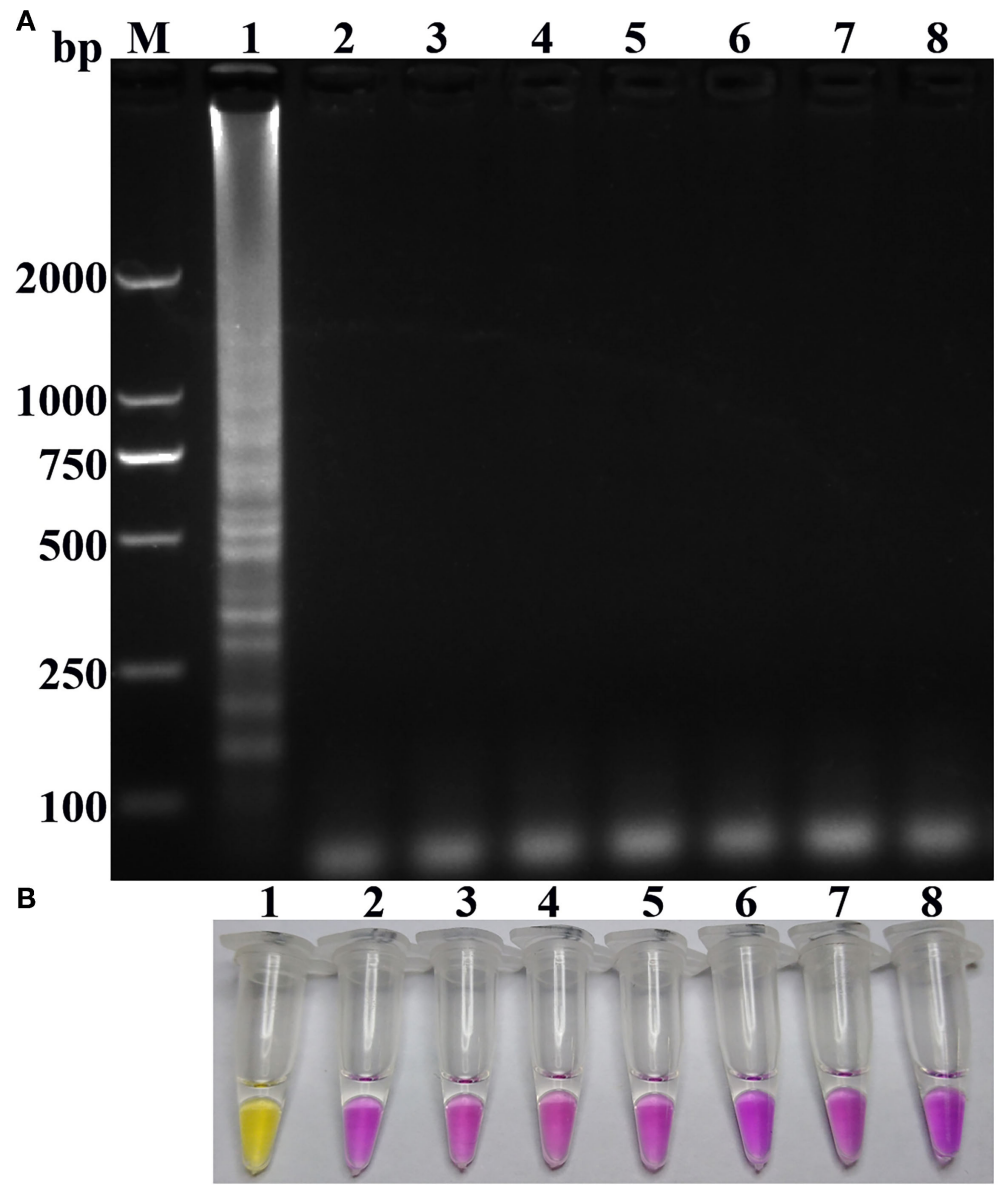
Specificity of the one-step RT-PSR amplification for N-GoAstV: **(A)** Agarose gel electrophoresis of RT-PSR amplification products. **(B)** Visual detection of positive and negative PSR amplification products. Lane M, molecular size marker DL2000; Lane 1, N-GoAstV; Lane 2, GPV; Lane 3, GHPV; Lane 4, GREOV; Lane 5, TMUV; Lane 6, goose-origin astrovirus FLX; Lane 7, goose-origin astrovirus AHDY; Lane 8, negative control.

As shown in Figure 2, the results of the specificity of the one-step RT-PSR as shown by gel electrophoresis and visualized by pH-sensitive color-changing dye, indicated that only N-GoAstV could be amplified as a template, and the other goose reference pathogens and nuclease-free water did not yield any amplified products during the one-step RT-PSR. Meanwhile, no positives were detected in all of the replicate tests, including no-template controls and off-target virus controls (95% CI).

### Clinical Sample Testing

All the samples (*n* = 235) were detected for the presence of N-GoAstV by RT-LAMP and one-step RT-PSR. The positive rates of RT-LAMP and one-step RT-PSR were 186/235 and 186/235, respectively (Table 2). The results demonstrated that the one-step RT-PSR assay was as accurate as the RT-LAMP in diagnosis of field N-GoAstV samples, and the presence of N-GoAstV RNA in these positive samples for one-step RT-PSR were also confirmed by PCR and sequencing.

**Table 2:** Comparison of virus isolation, RT-PSR and RT-LAMP for detection of N-GoAstV in clinical samples.

**Virus isolation**	**RT-LAMP**		**RT-PSR**		**Kappa (κ)**	***p*-value of Kappa**
	**Positive**	**Negative**	**Positive**	**Negative**		
Positive	186	0	186	0	1.000^*^	<0.001
Negative	0	49	0	49		

* *Agreement of results between RT-PSR and virus isolation*.

## Discussion

To the best of our knowledge, no commercial vaccines or effective therapeutic schedules have been set up to control the gout-related disease caused by N-GoAstV. Thus, establishment of a rapid and simple detection method is imperative. Although some PCR-based methods have been successfully used and have proven effective for N-GoAstV detection (5, 7, 10, 11), the complicated test procedures and expensive equipment required limited their extensive practical use. In the recent years, to circumvent the need for professional laboratory equipment, many detection methods including LAMP and PSR assays were invented for use under isothermal conditions (14–16, 19, 20). However, the RT-LAMP assay for N-GoAstV detection requires two primer pairs to target at least six sequence regions and strict primer coordination. Since the N-GoAstV sequences occur in many mutations, primers are easily mismatched resulting in failure to match with GoAstVs sequences (7). Compared with PCR and qPCR, PSR needs only a single pair of primers and no sophisticated equipment or professional technicians, resulting in cost savings and an extended range of application. Based on these advantages, PSR should offer a viable alternative method by which to detect N-GoAstVs both at point of care and on-site.

In this study, the PSR method was used to diagnose N-GoAstV and shown to be an excellent detection method for N-GoAstV. The primers designed were simpler in PSR than those in LAMP and were selective only in the target region of N-GoAstV in the same way as in conventional PCR primers. The optimum reaction conditions were temperature at 62°C and a time of 40 min. Hence, the reaction can be conducted in a water bath, meaning that the test is readily accessible. In established LAMP assays, Yu et al. used SYBR Green I dye to display a remarkable dissimilarity between positive and negative reactions, but the dye cannot be added before the reaction starts because of its inhibitory effect (21). This means the reaction must be uncovered in order to add the dye, increasing the risk of aerosol cross-contamination. SYTO 9, SYTO 82, SYTO 16, SYTO 13, and Miami Yellow—different from SYBR Green I, —were found to be the best dyes with no inhibitory effect in the real-time LAMP reactions (22). Whether these dyes in high concentration could be used for visible observation by the naked eye under natural light needs further evaluation. In this study, we used a different color indicator, including phenol red and cresol red, which had been developed for use in RT-LAMP assays as previously described (13). The specificity of the primers was verified by detecting the pathogenic agents N-GoAstV, GPV, GREOV, GHPV, and TMUV, and only the N-GoAstV produced a ladder pattern, indicating that the method established was highly specific. The practicability of the RT-PSR test for clinical diagnosis was determined by testing 235 samples from goose farms in the field and further confirmed by sequencing of the amplified products of positive samples. The RT-PSR test displayed perfect concordance between virus isolation and RT-LAMP. The high success rate may have been due to the high viral load of the 186 positive samples (Ct value by RT-qPCR performed on the same samples were all determined to be below 27) (13). Meanwhile, alignment of sequences of some products also indicated the conservation of the primer targeting region, which also supported the high success rate (Supplementary Figure 2). Moreover, referring to the development of the LAMP assay, performing RT-PSR with crude RNA preps (e.g., detergent or boiling lysis), would make it suitable for field use profit by Bst polymerase which theoretically be less affected by potential inhibitors within the samples (23). Meanwhile, a multiplexing-PSR assay using some kind of fluorophore and quencher reporter could be developed in a well-equipped laboratory since the number of primers is small.

## Conclusion

The RT-PSR established in this study provides an effective technique for rapid diagnosis of N-GoAstV in the field, which allows improved surveillance of the disease as well as facilitating investigation into the molecular epidemiology of GoAstV.

## Data Availability Statement

The original contributions presented in the study are included in the article/Supplementary Material, further inquiries can be directed to the corresponding author/s.

## Ethics Statement

The animal study was reviewed and approved by South China Agricultural University Committee for Animal Experiments (ID: SYXK-2014-0136).

## Author Contributions

JJ and XX designed the study and wrote the manuscript. QC, ZY, and XM performed the sampling and data collection. XT and XF performed the primer design and method optimum. YB, QX, and LY performed the clinical sample detection and helped to draft the manuscript. All authors have read and approved the final manuscript.

## Conflict of Interest

The authors declare that the research was conducted in the absence of any commercial or financial relationships that could be construed as a potential conflict of interest.

## Supplementary Material

Supplementary Figure 1 **(a)** One-step RT-PSR amplification results at different temperatures. Lane M, molecular size marker DL2000; Lane 1: 60°C; Lane 2: 61°C; Lane 3: 62°C; Lane 4: 63°C; Lane 5: 64°C; Lane 6: 65°C; Lane 7: negative control. **(b)** One-step RT-PSR amplification results at different reaction time. Lane M, molecular size marker DL2000; Lane 1: 10 min; Lane 2: 20 min; Lane 3: 30 min; Lane 4: 40 min; Lane 5: 50 min; Lane 6: 60 min; Lane 7: negative control.

Supplementary Figure 2 Alignment of some sequence of the products of positive clinical samples. Sequence highlighted with a black background indicates primer target region. Identical nucleotide acids are marked with red above.

Supplementary Table 1 Assay data used for probit analysis to calculate the detection limits of N-GoAstV by one-step RT-PSR assay.
