# Supplementary figures and images for: Simple and Visible Detection of Novel Astroviruses Causing Fatal Gout in Goslings Using One-Step Reverse Transcription Polymerase Spiral Reaction Method
Source: Front Vet Sci. 2020 Dec 10;7:579432. doi: 10.3389/fvets.2020.579432 (PMC7758545; doi:10.3389/fvets.2020.579432)

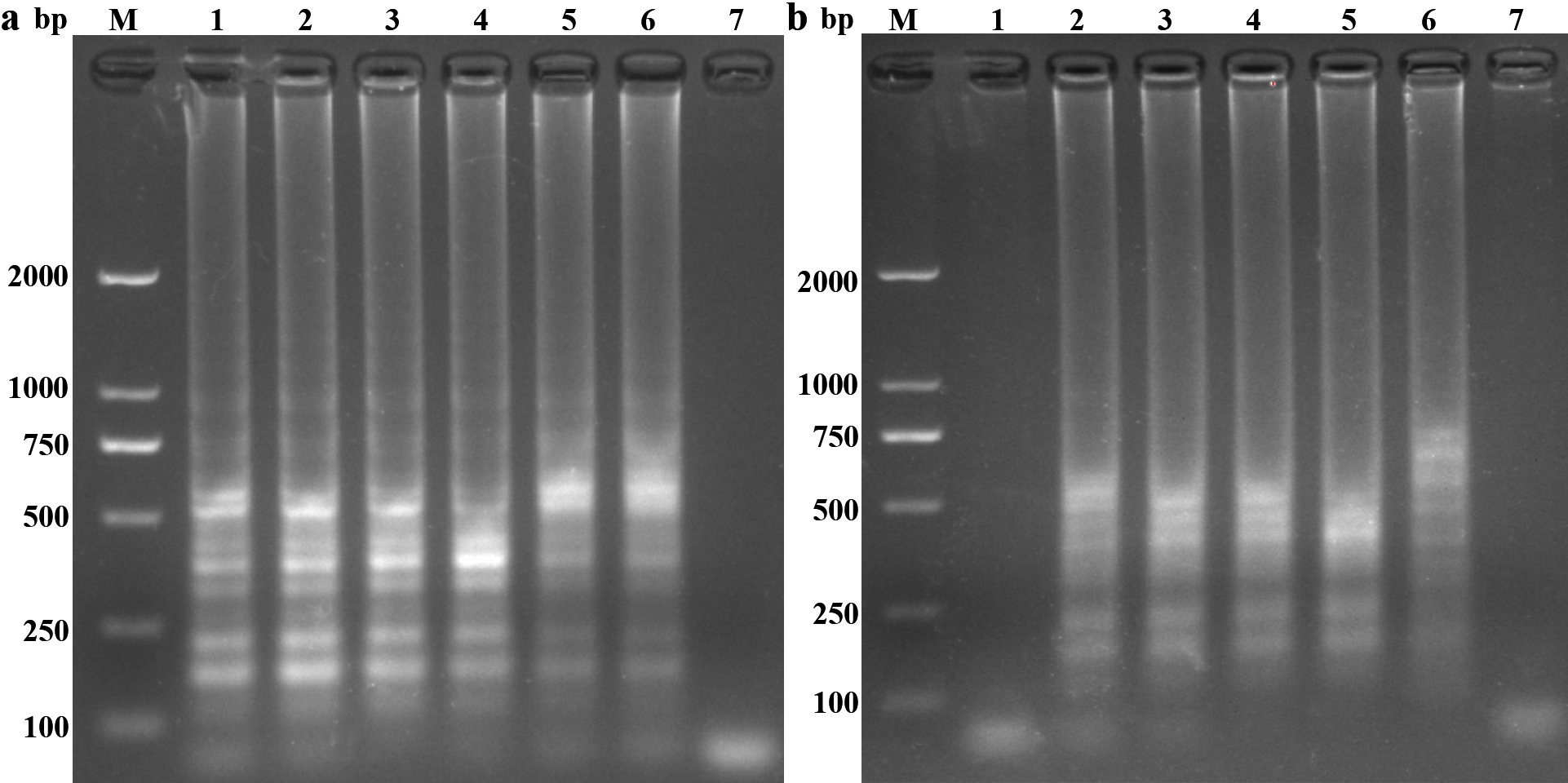

Supplement: Supplementary Figure 1 — (a) One-step RT-PSR amplification results at different temperatures. Lane M, molecular size marker DL2000; Lane 1: 60°C; Lane 2: 61°C; Lane 3: 62°C; Lane 4: 63°C; Lane 5: 64°C; Lane 6: 65°C; Lane 7: negative control. (b) One-step RT-PSR amplification results at different reaction time. Lane M, molecular size marker DL2000; Lane 1: 10 min; Lane 2: 20 min; Lane 3: 30 min; Lane 4: 40 min; Lane 5: 50 min; Lane 6: 60 min; Lane 7: negative control. [file Image_1.TIF]

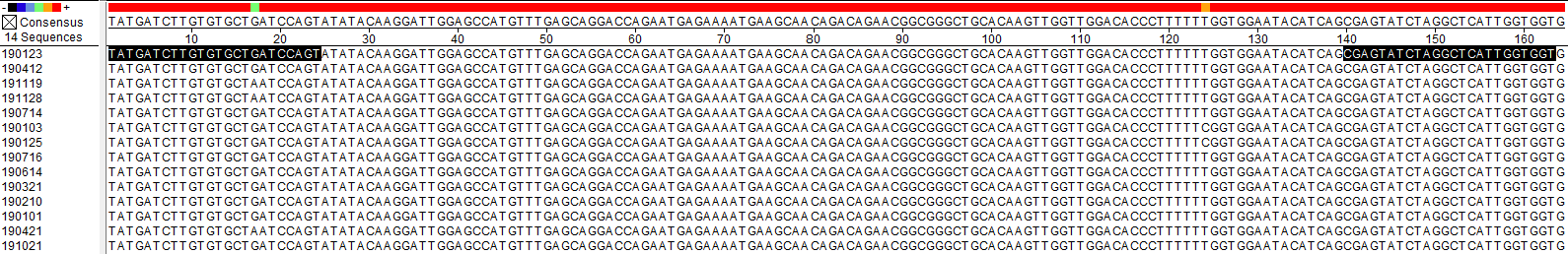

Supplement: Supplementary Figure 2 — Alignment of some sequence of the products of positive clinical samples. Sequence highlighted with a black background indicates primer target region. Identical nucleotide acids are marked with red above. [file Image_2.TIF]
