# Supplementary material for: Simple and Visible Detection of Novel Astroviruses Causing Fatal Gout in Goslings Using One-Step Reverse Transcription Polymerase Spiral Reaction Method
Source: Front Vet Sci. 2020 Dec 10;7:579432. doi: 10.3389/fvets.2020.579432 (PMC7758545; doi:10.3389/fvets.2020.579432)
Supplement: Supplementary Table 1 — Assay data used for probit analysis to calculate the detection limits of N-GoAstV by one-step RT-PSR assay. [file Table_1.DOCX]

Supplementary Table 1. Assay data used for probit analysis to calculate the detection limits of N-GoAstV by one-step RT-PSR assay

| Copies per reaction | No. of positive samples  / No. of samples |
| --- | --- |
| 3.47 × 10^6^ | 20/20 |
| 3.47 × 10^5^ | 20/20 |
| 3.47 × 10^4^ | 20/20 |
| 3.47 × 10^3^ | 20/20 |
| 3.47 × 10^2^ | 20/20 |
| 3.47 × 10^1^ | 20/20 |
| 3.47 | 1/20 |
